# Ectodermal dysplasia-related disorders of the respiratory tract

**DOI:** 10.1186/1746-160X-8-S1-I16

**Published:** 2012-05-25

**Authors:** Timothy J Fete

**Affiliations:** 1 University of Missouri Department of Child Health, Columbia, USA

## 

Ectodermal dysplasia syndromes are frequently associated with manifestations of disease in the respiratory tract. Commonly, patients present with signs and symptoms of sinusitis, otitis media with effusion, bronchitis or pneumonia. The etiologic bases for these disorders are multi-factorial, including abnormal mucous production, anatomical variations, atopy and immune function abnormalities. This presentation reviews the current literature on the clinical manifestations and potential underlying causes for the noted increase in morbidity due to respiratory disease in the ectodermal dyplasia syndromes.

